# Effect of Sodium Butyrate Supplementation on Type 2 Diabetes—Literature Review

**DOI:** 10.3390/nu17111753

**Published:** 2025-05-22

**Authors:** Wiktoria Krauze, Nikola Busz, Weronika Pikuła, Martyna Maternowska, Piotr Prowans, Dominika Maciejewska-Markiewicz

**Affiliations:** 1 Department of Human Nutrition and Metabolomics, Pomeranian Medical University, 71-460 Szczecin, Poland; wiktoria.krauze00@gmail.com (W.K.); busz.dietetyka@gmail.com (N.B.); weronika.pikula01@o2.pl (W.P.); maartyna.jr@gmail.com (M.M.); 2 Department of Plastic, Endocrine and General Surgery, Pomeranian Medical University, 72-010 Szczecin, Poland; piotr.prowans@pum.edu.pl

**Keywords:** type 2 diabetes mellitus, gut microbiota, intestinal permeability, butyrate, sodium butyrate supplementation, metabolic endotoxemia, inflammation, glucose metabolism, insulin resistance

## Abstract

**Background:** Type 2 diabetes mellitus (T2DM) represents a major global health burden, with prevalence rates escalating due to rapid urbanization, economic growth, and the obesity epidemic. Despite intensive research, the underlying molecular mechanisms remain incompletely understood, with emerging evidence suggesting multifactorial origins involving genetic, epigenetic, lifestyle, and environmental factors. **Methods:** This review synthesizes current epidemiological data on T2DM prevalence, risk factors, and demographic patterns from 1990 to 2017, and discusses projected trends through 2030. We examine the role of intestinal barrier dysfunction and gut microbiota dysbiosis in T2DM pathogenesis, highlighting key mechanistic insights. Furthermore, we analyze recent findings on the role of butyrate, a major short-chain fatty acid, in preserving gut integrity and its potential therapeutic effects on metabolic health. **Results:** Global T2DM prevalence has risen markedly across all age groups, with particularly high rates in Western Europe and Pacific Island nations. Disruption of the intestinal barrier (“leaky gut”) and gut microbiota alterations contribute significantly to systemic inflammation and insulin resistance, which are pivotal features in T2DM development. Butyrate plays a central role in maintaining epithelial barrier function, modulating immune responses, and regulating glucose metabolism. Preclinical studies have demonstrated that sodium butyrate supplementation improves gut integrity, reduces systemic endotoxemia, and ameliorates metabolic parameters. Emerging clinical evidence suggests benefits of sodium butyrate, particularly when combined with prebiotic fibers, in improving glycemic control and reducing inflammatory markers in T2DM patients. **Conclusions:** Gut barrier integrity and microbiota composition are critical factors in T2DM pathogenesis. Sodium butyrate shows promise as a complementary therapeutic agent in T2DM management, although further large-scale, long-term clinical trials are required to confirm its efficacy and safety. Targeting gut health may represent a novel strategy for the prevention and treatment of T2DM.

## 1. Pathogenesis of Type 2 Diabetes

Type 2 diabetes mellitus (T2D) constitutes a significant public health concern, substantially impacting both patient life expectancy and healthcare expenditures. The global prevalence of this condition is steadily increasing, a trend closely associated with rapid economic growth and urbanization [1]. The primary clinical manifestation of T2D is hyperglycemia, which has emerged as one of the fastest-growing global health threats in the 21st century. This chronic and progressive disease develops due to the body’s inability to effectively utilize insulin or insufficient insulin production, and its rising incidence is inextricably linked to the worldwide obesity epidemic [2]. Despite extensive research efforts, the precise molecular mechanisms underlying the pathogenesis of type 2 diabetes remain incompletely understood. Despite that impaired insulin secretion is the only prerequisite for T2DM development, mounting evidence suggests a multifactorial etiology involving lifestyle, environmental exposures, genetic predisposition, and epigenetic modifications [3]. Obesity is a major environmental risk factor associated with T2D, as confirmed by numerous cross-sectional and prospective studies. Approximately 50% of individuals with type 2 diabetes are obese (body mass index (BMI) > 30 kg/m^2^), while nearly 90% are overweight (BMI > 25 kg/m^2^), indicating that even modest weight reduction may lead to significant improvements in disease management and metabolic control [4].

Furthermore, a sedentary lifestyle has been shown to substantially increase the risk of developing T2D, primarily through mechanisms involving reduced insulin sensitivity and impaired glucose uptake [5]. Advancing age is also associated with an increased risk, likely due to age-related declines in insulin sensitivity [6]. Smoking has been identified as another modifiable risk factor. One study reported that individuals who smoke 20 cigarettes per day exhibit a 61% higher risk of developing T2D, while those with lower levels of tobacco consumption have a 29% increased risk. Among individuals with normal BMI, smokers were found to have higher levels of abdominal adiposity compared to non-smokers—a major risk factor for T2D [7,8]. Alcohol consumption may also contribute to the risk of developing type 2 diabetes. Epidemiological evidence suggests that intake exceeding 63 g of alcohol per day may correlate with a higher incidence of the disease [9].

Genetic susceptibility represents another critical risk factor. Several studies have demonstrated that certain ethnic groups are disproportionately affected by T2D. For instance, Pima Indians residing in Western countries are twice as likely to develop the disease compared to individuals of European descent. Moreover, individuals with a parental history of T2D have a sixfold increased risk of developing the condition relative to those without such a family history [10].

## 2. Global Trends and Demographic Patterns of T2D

T2D spanning the period from 1990 to 2017 reveal a substantial and sustained global increase in disease prevalence across all age groups. In 2017, approximately 462 million individuals were living with T2D, representing 6.28% of the global population. Stratified by age, the prevalence was 4.4% among individuals aged 15–49 years, 15% among those aged 50–69 years, and 22% in individuals aged 70 years and older. This equates to a global prevalence rate of 6059 cases per 100,000 population [11]. While the burden of T2D is rising globally, the rate of increase is notably more pronounced in high-income regions, particularly Western Europe. Diabetes currently accounts for approximately 1 million deaths annually, positioning it as the ninth leading cause of mortality worldwide. The disease significantly contributes to premature mortality through its association with reduced quality of life, diminished functional capacity, and elevated risk of multiple comorbid conditions [11,12].

Projections indicate that by the year 2030, the global prevalence of type 2 diabetes will rise to approximately 7079 cases per 100,000 population, reflecting a consistent upward trajectory across all world regions. Within Europe, the prevalence is estimated at 8529 cases per 100,000, with the highest national rates observed in countries such as Germany, the Netherlands, Switzerland, Italy, and Sweden [12]. Interestingly, some of the most elevated prevalence rates are found in specific island nations of the Pacific Ocean. Notable examples include American Samoa (18,312 cases per 100,000), Kiribati (17,432), Mauritius (18,545), and Fiji (20,277). In terms of absolute numbers, countries with large populations such as the United States (28.9 million cases), India (65.9 million), and China (88.5 million) rank highest globally in the total number of individuals affected by type 2 diabetes [11]. Recent research highlights that over one-third of diabetes-related deaths occur in individuals under the age of 60, underscoring the premature mortality associated with the disease [12].

Epidemiological patterns also reveal that men are diagnosed with T2D more frequently than women. However, the principal modifiable risk factor—obesity—is more prevalent among women. This sex-based disparity may be attributed to a complex interplay of biological, behavioral, psychosocial, socioeconomic, cultural, environmental, anatomical, physiological, genetic, and epigenetic factors [12]. Moreover, type 2 diabetes manifests more aggressively in adolescent populations than in adults. This is largely due to the early onset of comorbidities and complications, including hypertension, nephropathy, polycystic ovary syndrome (PCOS), and dyslipidemia, which accelerate the disease course and increase its severity in youth [13,14].

## 3. Disruption of the Intestinal Barrier and Its Implications in Type 2 Diabetes Mellitus

The gut microbiota, also referred to as the microbiome, comprises a complex community of microorganisms that inhabit the human gastrointestinal tract and engage in dynamic interactions with the host. It plays a pivotal role in modulating host immunity, regulating metabolic pathways, and maintaining intestinal homeostasis [15,16]. Colonization of the gut begins within hours after birth and continues throughout an individual’s lifetime. In healthy adults, over 90% of the gut microbiota is composed of bacterial species from the phyla *Bacteroidetes* and *Firmicutes* [16,17,18]. The composition of the intestinal microbiota is influenced by a multitude of factors, including age, dietary patterns, obesity, physical activity levels, ethnicity, psychological stress, smoking, alcohol consumption, and pharmacological treatments. Alterations in these factors can lead to dysbiosis—a state of microbial imbalance characterized by quantitative and qualitative changes in microbial populations—ultimately contributing to impaired physiological function and disease susceptibility [16,19,20,21].

Extensive research has established a link between gut microbiota dysregulation and the pathophysiology of several chronic diseases, notably type 2 diabetes mellitus (T2DM) [22]. T2DM is a metabolic disorder marked by impaired insulin secretion and disrupted carbohydrate metabolism, leading to chronic hyperglycemia. T2DM is predominantly associated with unhealthy lifestyle factors, particularly long-term consumption of energy-dense, nutrient-poor diets. Such diets disrupt the gut microbiota by reducing the abundance of fiber-degrading and barrier-supporting bacterial species—including *Lactobacillus*, *Bacillus*, *Bifidobacterium*, *Faecalibacterium prausnitzii*, *Roseburia*, and *Bacteroides vulgatus*—and promoting the overgrowth of mucus-degrading and potentially pathogenic bacteria such as *Akkermansia muciniphila*, *Bacteroides caccae*, *Clostridium*, and *Escherichia coli* [23,24,25,26]. The dysbiosis is accompanied by a disturbed *Bacteroidetes*/*Firmicutes* ratio and contributes to the phenomenon commonly referred to as “leaky gut” [27]. Increased intestinal permeability allows for the translocation of microbial products, most notably bacterial lipopolysaccharides (LPS)—components of Gram-negative bacterial membranes—into systemic circulation [21,27,28]. This process elicits a systemic inflammatory response and activates the immune system, contributing to the exacerbation of microvascular complications commonly seen in diabetes [21,23,29,30]. Moreover, increased intestinal permeability is associated with elevated expression of zonulin, a regulator of tight junctions. Zonulin modulates the epithelial barrier function and initiates immune responses, including the release of pro-inflammatory cytokines that compromise epithelial integrity [29,30,31]. Studies in diabetic murine models have confirmed the presence of metabolic endotoxemia and compromised gut barrier function [21].

**Figure 1:**
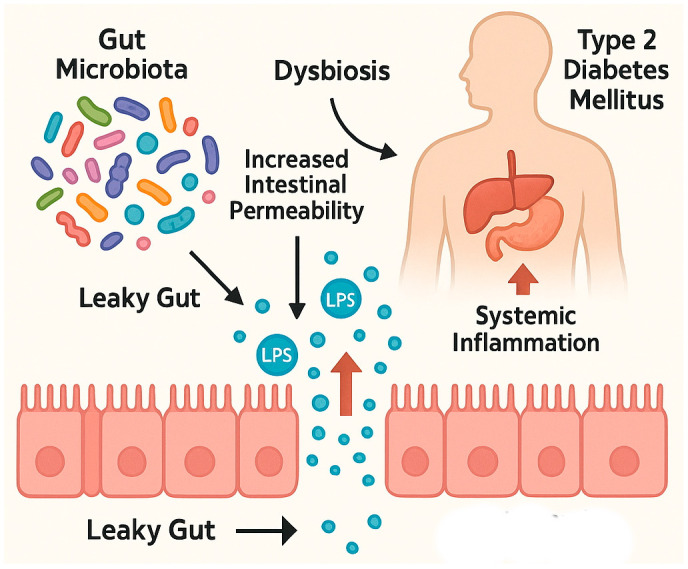
Disruption of the intestinal barrier in T2D. Created with Biorender and ChatGPT 4.0.

One of the downstream consequences of intestinal permeability is in increase in metabolic imbalance [23]. Research by Cani et al. demonstrated that prebiotic supplementation could restore intestinal integrity in diabetic mice, suggesting a therapeutic avenue for mitigating T2DM-associated complications [32]. Similar results have been observed in clinical studies involving human subjects with T2DM [21]. Indeed, a 64% elevation in fasting plasma LPS concentrations was reported in individuals with T2DM compared to healthy controls [21,28]. Increased gut permeability to LPS has been implicated in the maintenance of chronic low-grade inflammation and reduced insulin sensitivity, both of which are central to the pathogenesis of T2DM [26]. The human intestine lacks the enzymes necessary to digest certain complex carbohydrates from the fiber, which are instead fermented by the enteric microbiome to short-chain fatty acids (SCFAs). Individuals with T2DM exhibit significantly lower concentrations of SCFAs —notably, acetate, propionate, lactate, and butyrate—produced predominantly in the colon through microbial fermentation [26,33]. This reduction is attributed to a shift in gut microbiota composition, where fiber-degrading, SCFA-producing anaerobes diminish due to decreased fiber intake and elevated luminal pH. In response, mucus-degrading bacteria proliferate, further compromising the gut barrier. The resulting dysbiosis leads to increased LPS levels, systemic inflammation, and impaired glucose metabolism, including reduced secretion of incretin hormones such as glucagon-like peptide-1 (GLP-1) and peptide YY [25,26,33,34]. Furthermore, individuals with T2DM have been found to exhibit significantly elevated fecal zonulin levels compared to healthy individuals, providing direct evidence of compromised intestinal barrier integrity in this population [25]. Disruption of the intestinal barrier in T2D was shown in Figure 1.

## 4. Mechanism of Butyrate Formation in the Gastrointestinal Tract

Butyric acid (butyrate) constitutes approximately 15–23% of the total pool of short-chain fatty acids (SCFAs) detected in human fecal samples. Its production is contingent upon both the type and quantity of dietary fiber consumed, as well as the presence of gut microbiota with the metabolic capacity to synthesize it [35]. To date, over 50 bacterial genera and approximately 400 species have been identified in human stool, with obligate anaerobes—such as *Bacteroides*, *Bifidobacteria*, *Eubacteria*, *Streptococci*, and *Lactobacilli*—comprising the dominant microbial populations [36]. Facultative anaerobic species, including various *Enterobacteriaceae*, are also present, albeit in lower abundance. The principal butyrate-producing bacteria in the human gastrointestinal tract belong to the phylum *Firmicutes*, particularly *Faecalibacterium prausnitzii* and *Clostridium leptum* (family *Ruminococcaceae*), as well as *Eubacterium rectale* and species of the genus *Roseburia* (family *Lachnospiraceae*) [37]. Other butyrate-producing bacteria, such as *Eubacterium hallii* and *Anaerostipes* spp., utilize lactate and acetate as substrates to generate butyrate [38].

It is important to note that the catalog of known butyrate-producing taxa is likely incomplete. Additional potential producers have been identified across various phyla, including *Actinobacteria*, *Bacteroidetes*, *Fusobacteria*, *Proteobacteria*, *Spirochaetes*, and *Thermotogae* [39]. Furthermore, bacteria of the genus *Bifidobacterium* have also demonstrated butyrogenic capacity [40]. A recent study suggests that *Akkermansia muciniphila* may indirectly elevate butyrate concentrations near the intestinal epithelium, conferring potential health benefits to the host [41].

**Figure 2:**
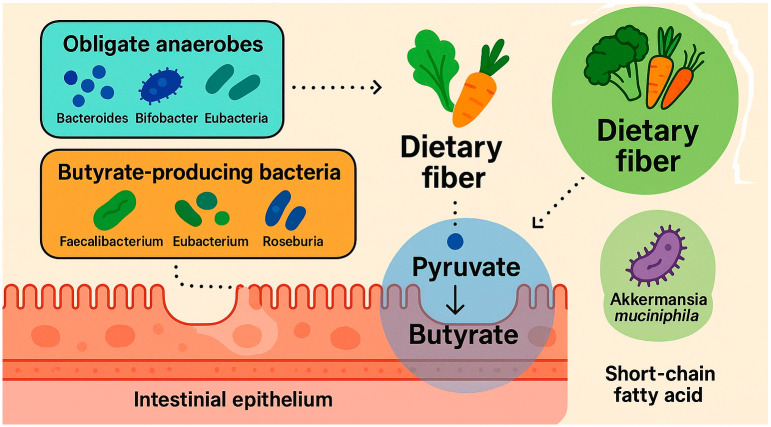
Mechanism of butyrate formation in the gastrointestinal tract. Created with Biorender and ChatGPT 4.0.

The primary metabolic pathway for butyrate synthesis in the gut involves the conversion of pyruvate—derived from carbohydrate fermentation—into butyrate. Alternative metabolic pathways, such as those utilizing lysine, glutarate, or 4-aminobutyrate (GABA), contribute to a lesser extent and are typically associated with the metabolism of dietary proteins. The pyruvate/acetyl-CoA pathway is estimated to be present in approximately 24% of gut bacteria, whereas the protein-derived pathways are found in less than 8% of microbial taxa [35,39]. Two main metabolic routes facilitate microbial butyrate synthesis in the colon. In the first, fermentation-derived butyryl-CoA is converted to butyryl-phosphate, which is subsequently transformed into butyrate by the enzyme butyrate kinase. In the second route, the enzyme butyryl-CoA:acetate CoA-transferase mediates the transfer of the CoA moiety from butyryl-CoA to acetate, yielding butyrate and acetyl-CoA as products [39]. Metagenomic analyses further suggest that butyrate can also be generated via the lysine fermentation pathway from protein substrates [42]. Additionally, exogenous sources of butyrate include certain dietary components—most notably dairy fats, which contain tributyrin (a triglyceride form of butyrate), contributing 3–8% of total fatty acid content [35]. Mechanism of butyrate formation in the gastrointestinal tract are shown in Figure 2.

## 5. Forms, Dosages, and Safety of Sodium Butyrate Supplementation

Sodium butyrate is a promising dietary supplement with a broad range of therapeutic applications, particularly in the management of intestinal and metabolic disorders. It is available in various formulations, including capsules, tablets, powders, and microencapsulated preparations, with the latter offering the most effective targeted delivery to the intestine [43].

The appropriate dose of sodium butyrate depends on the therapeutic indication, patient age, and the formulation used. However, dosages reported in the literature vary considerably. For example, in a study by Facchin S et al. [43], patients with inflammatory bowel disease (IBD) received an oral microencapsulated sodium butyrate formulation (Butyrose^®^ Lsc Microcaps-EP2352386B1, BLM, Sila Srl, Noale, Italy) at a dose of 1800 mg/day (three capsules daily) for 60 days. The control group received starch capsules identical in color, taste, and size. A total of 49 IBD patients (n = 19 Crohn’s disease [CD]; n = 30 ulcerative colitis [UC]) were included. At baseline, healthy volunteers exhibited a distinct microbiota composition compared to IBD patients. Sodium butyrate supplementation promoted the growth of short-chain fatty acid (SCFA)-producing bacteria, specifically Lachnospiraceae spp. in UC patients and *Butyricicoccus* spp. in CD patients, suggesting an anti-inflammatory potential [43].

In another study, hypertensive patients received oral sodium butyrate at a daily dose of 3.9 g for four weeks, while the control group received a placebo containing an equivalent amount of sodium chloride (2.0 g). Both groups experienced an additional sodium load of 798 mg daily. Results indicated that oral butyrate supplementation was associated with an increase in daytime systolic and diastolic blood pressure, highlighting a potential adverse effect in hypertensive populations and underscoring the need for caution [44].

Conversely, in a large observational study involving 3000 non-hospitalized patients with confirmed irritable bowel syndrome (IBS), supplementation with sodium butyrate in a triglyceride matrix (150 mg twice daily for 12 weeks) was associated with symptom improvement and enhanced quality of life [45]. Another randomized controlled trial assessed the effects of microencapsulated sodium butyrate (1.5 g/day for 12 weeks) in 52 patients with type 2 diabetes mellitus (T2DM) and abdominal pain. A significantly greater proportion of patients in the butyrate group reported relief from gastrointestinal symptoms, along with a reduced incidence of small intestinal bacterial overgrowth (SIBO), and slight but significant improvements in BMI and glycated hemoglobin (HbA1c) levels [46].

Furthermore, sodium butyrate supplementation has been investigated in pediatric populations. A study on children with obesity administered sodium butyrate (20 mg/kg/day, up to a maximum of 800 mg/day) in addition to standard care for six months. The primary outcome—a reduction of at least 0.25 standard deviations in BMI—was achieved, alongside improvements in waist circumference, glucose metabolism, lipid profiles, inflammatory markers, and gut microbiota composition. Mild and transient adverse events, such as nausea and headache, were reported by only two participants during the first month of treatment [47].

In summary, sodium butyrate appears to be a safe and well-tolerated supplement, with infrequent and mild side effects. Its supplementation shows particular benefits in patients with IBS, metabolic disorders (such as obesity and T2DM), and IBD. However, caution is warranted in hypertensive individuals, given the potential for increased blood pressure with prolonged use due to additional sodium intake. Clinical use of sodium butyrate should be individualized, carefully selected based on the patient’s condition, and conducted under the supervision of a physician or clinical nutritionist. Further research is needed to better define optimal dosing strategies for specific disease states and to elucidate the long-term safety profile of sodium butyrate supplementation.

## 6. Effect of Butyrate on the Intestinal Microbiota

Butyrate, a key metabolite among short-chain fatty acids (SCFAs), plays a crucial role in maintaining intestinal health. It serves as a primary energy source for colonocytes and supports the integrity of the intestinal epithelium. Butyrate is predominantly produced by members of the Clostridium cluster within the Firmicutes phylum, including *Faecalibacterium*, *Eubacterium*, *Anaerostipes*, *Roseburia*, *Coprococcus*, *Anaerobutyricum*, and *Subdoligranulum* [48]. The presence of butyrate-producing bacteria helps sustain an anaerobic environment in the gut, preventing colonization by opportunistic aerobic pathogens such as *Escherichia coli* [49].

The majority of colonic butyrate is generated through the fermentation of dietary fibers, primarily of carbohydrate origin. As a weak acid (pKa = 4.8), butyrate is absorbed via two principal transporters: the sodium-coupled monocarboxylate transporter 1 (SMCT1, encoded by *SLC5A8*) and the proton-coupled monocarboxylate transporter 1 (MCT1, encoded by *SLC16A1*) [35]. Approximately 70% of the absorbed butyrate is utilized for energy production through the citric acid cycle. The remaining butyrate enters the portal circulation, serving as an energy source for hepatocytes before reaching systemic tissues [48]. SMCT1, characterized by high affinity for butyrate, is predominantly expressed in the distal colon where butyrate concentrations are lower, promoting concurrent sodium and water absorption. In contrast, MCT1, with lower substrate affinity, is highly expressed in the proximal colon and facilitates butyrate uptake in a proton-dependent manner [50,51].

Once internalized, butyrate activates G-protein-coupled receptors (GPRs) such as GPR109A (hydroxycarboxylic acid receptor 2; HCAR2), GPR41 (free fatty acid receptor 3; FFAR3), and GPR43 (free fatty acid receptor 2; FFAR2), expressed on intestinal epithelial cells [52].

Both endogenous and orally administered butyrate exert multidimensional effects on intestinal health [53]. Through GPR activation, butyrate modulates immune responses in the colonic mucosa, influencing neutrophil, macrophage, and epithelial cell activity. It stimulates the release of gastrointestinal hormones such as glucagon-like peptide-1 (GLP-1), GLP-2, and peptide YY (PYY), thereby impacting insulin sensitivity and gut motility regulation [54,55]. As a ligand for SCFA receptors, butyrate induces PYY secretion [56] and promotes serotonin release from enterochromaffin cells, further supporting intestinal motility [57].

Additionally, butyrate strengthens the intestinal barrier by modulating tight junction proteins. It downregulates claudin-2 expression via IL-10 receptor alpha (IL-10RA)-dependent pathways, reducing paracellular permeability and maintaining epithelial tension [58]. Butyrate also restores the tight junction protein Zonula Occludens-1 (ZO-1) complex, alleviating inflammation and barrier dysfunction [59]. Upregulation of synaptopodin (SYNPO), an actin-binding protein at tight junctions, further contributes to barrier integrity [60].

Butyrate exerts potent anti-inflammatory effects by inhibiting nuclear factor-kappa B (NF-κB) activation and histone deacetylase (HDAC) enzymes in both immune and epithelial cells [61]. By modulating histone acetylation and blocking NF-κB signaling, butyrate reduces colonic inflammation and suppresses the production of pro-inflammatory cytokines such as IL-1β, IL-2, IL-6, IL-8, IL-12, and TNF-α, as well as interferon-gamma-inducible protein 10 (IP-10) in colonic myofibroblasts [62,63]. Moreover, sodium butyrate supplementation mitigates ethanol-induced intestinal dysbiosis, epithelial barrier disruption, and increased permeability by enhancing tight junction repair [64].

In the large intestine, butyrate also promotes sodium (Na^+^) and chloride (Cl^−^) absorption, which enhances water reabsorption. This occurs through activation of ion transporters, particularly the Na^+^/H^+^ exchanger and the Na^+^/Cl^−^ cotransporter, facilitating electrolyte and water balance [65]. Furthermore, butyrate supports intestinal mucosal health by increasing the expression of the *MUC2* gene family, thus stimulating protective mucus synthesis and reinforcing the gut–blood barrier [66]. Importantly, reduced butyrate levels are associated with decreased abundance of tight junction proteins in the proximal colon, oxidative stress, hepatic steatosis, and inflammation [67].

## 7. Effect of Sodium Butyrate in Type 2 Diabetes: A Study Overview

There is growing evidence supporting the pivotal role of butyrate and its interaction with insulin-secreting β-cells. The regulation of gluconeogenesis through histone deacetylase (HDAC) inhibition and the potent stimulation of glucagon-like peptide-1 (GLP-1)-mediated insulin secretion highlight butyrate as a promising candidate for diabetes therapy. Recent findings suggest that sodium butyrate supplementation may enhance insulin secretion indirectly by modulating the expression of key functional genes in β-cells isolated from rat pancreatic islets [68].

Animal model studies have demonstrated that butyrate supplementation results in reduced plasma glucose levels, lower HbA1c, and decreased total cholesterol and low-density lipoprotein (LDL) concentrations. In both experimental settings, butyrate exerted a protective effect on pancreatic β-cells and mitigated inflammation-induced functional impairment. These findings suggest that butyrate may attenuate insulin resistance, dyslipidemia, and gluconeogenesis, thereby contributing to improved glucose homeostasis [69].

Furthermore, data from studies in *db*/*db* mice models indicate that butyrate supplementation may alleviate diabetic endotoxemia by restoring gut microbiota composition and preserving intestinal epithelial barrier integrity. Collectively, these observations position sodium butyrate (NaBut) as a promising agent for the prevention and treatment of T2DM and associated dyslipidemia [70].

However, despite these encouraging preclinical results, clinical studies evaluating the efficacy of sodium butyrate in patients with type 2 diabetes remain limited and inconclusive. Further well-designed human trials are needed to fully elucidate its therapeutic potential (Table 1).

In a study by Roshanravan et al. [71], the effects of daily sodium butyrate (NaBut) and high-potency inulin (HP inulin) supplementation, administered individually and in combination, were evaluated in patients with type 2 diabetes mellitus (T2DM). The researchers assessed the impact of supplementation on glycemic control, glucagon-like peptide 1 (GLP-1) levels, and lipid profiles. Supplementation significantly reduced waist and hip circumferences in the groups receiving inulin and butyrate combined with inulin. Additionally, reductions in diastolic blood pressure were observed across all supplemented groups (butyrate, inulin, and butyrate + inulin). Notably, only the combination of butyrate and inulin led to a significant decrease in fasting blood glucose (FBS) levels, accompanied by a downward trend in HOMA-IR indices. Butyrate and butyrate + inulin supplementation significantly elevated postprandial GLP-1 concentrations compared to placebo; however, no significant changes in lipid profiles were noted. These findings suggest that combined supplementation of butyrate and inulin exerts beneficial effects on glycemic parameters and body composition in patients with T2DM [71]. The same study also examined the effects of supplementation on the expression of genes associated with pyroptosis, a form of programmed cell death activated in diabetes. Sodium butyrate significantly reduced the expression of *miR-146a-5p* and *miR-9-5p*, indicating its potential to mitigate pyroptosis by targeting the Toll-like receptor 2 (TLR2) and nuclear factor-kappa B1 (NF-κB1) pathways. These findings support the concept that butyrate, especially when combined with inulin (which enhances intestinal butyrate levels through fermentation), may modulate immune responses and oxidative stress, offering therapeutic potential in diabetes management [71].

Further, Roshanravan et al. [72] investigated the influence of sodium butyrate and HP inulin supplementation on the abundance of *Akkermansia muciniphila*, a key gut bacterium associated with metabolic health, and on the expression of Krüppel-like factor 5 (KLF5) and microRNA-375 (miR-375). Supplementation with HP inulin and butyrate increased the abundance of *A. muciniphila*, known for its anti-inflammatory and gut barrier-supporting properties [72]. Moreover, an upregulation of miR-375 was observed in the butyrate and butyrate + inulin groups compared to placebo, suggesting an additional mechanism by which these supplements may enhance gut and metabolic health [73].

A 2022 study further explored the metabolic effects of oral sodium butyrate supplementation in T2DM patients, assessing parameters such as blood pressure, oxidative stress markers (nitric oxide [NO] and glutathione peroxidase [Gpx]), and glycemic control [74]. After six weeks of supplementation, significant reductions in systolic and diastolic blood pressure were observed. Although postprandial blood glucose levels (BS2hpp) decreased significantly within both the intervention and placebo groups, intergroup differences were not statistically significant. While sodium butyrate significantly increased insulin levels and total cholesterol, and lowered NO levels, no significant differences between groups were detected. Additionally, no adverse biochemical changes were noted [74].

The most recent study conducted in 2024 [46] evaluated the effects of microencapsulated sodium butyrate (1.5 g/day) administered for 12 weeks to 52 patients with T2DM experiencing significant abdominal pain. This represented the highest dose of sodium butyrate administered to date in T2DM clinical studies. Patients receiving butyrate supplementation experienced significant improvements in gastrointestinal symptoms, BMI, and HbA1c levels compared to the placebo group [46].

**Table 1:** The impact of sodium butyrate intake on type 2 diabetes in animal and human models.

**References**	**Model**	**Study Groups**	**Intervention**	**Survey Results**
S. Khan et al. (2016) [69]	Animals	Sprague-Dawley rats with type 2 diabetes (n = 34)	NaB doses of 200 and 400 mg/kg twice daily or control metformin 150 mg/kg twice; for 10 weeks	Butyrate significantly reduced plasma glucose, HbA1c, insulin resistance, gluconeogenesis, and dyslipidaemia comparably to metformin. Butyrate alleviated micro- and macro-vesicular steatosis of the liver and fat deposition in brown adipose tissue and white adipose tissue (adipocyte hypertrophy). Butyrate also reduced damage to pancreatic beta cells.
Y. Hu et al. (2018) [75]	Animals	Sprague-Dawley rats with type 2 diabetes (n = 40)	NaB (500 mg/kg/d) was injected intraperitoneally; for 6 weeks	NaB improved insulin resistance and β-cell function and reduced β-cell apoptosis in rats with T2DM. NaB alleviated hyperglycaemia, lowered TC and LDL-c levels, prevented weight loss, and increased glucose tolerance. NaB ameliorated diabetes-induced islet histological changes and functional damage and alleviated β-cell apoptosis.
Ty-Hua Xu et al. (2018) [70]	Animals	7-week-old male *db*/*db* mice (type 2 diabetes; n = 24)	(1) model group (2) intervention group (sodium butyrate 0.5 g/kg/day); (3) Metformin 0.15 g/kg/day control group);	Butyrate significantly reduced blood HbA1c, inflammatory cytokines, and LPS levels in *db*/*db* mice. NaB reduced inflammatory cell infiltration and increased intestinal integrity and intercellular adhesion molecules. NaBut had the effect of reducing the Firmicutes:Bacteroidetes ratio. In Caco-2 cells, butyrate significantly promoted cell proliferation, enhanced the cells’ ability to counteract oxidative stress, reduced the secretion of inflammatory cytokines, and preserved the single-cell integrity of the epithelium.
N. Roshanravan et al. (2017) [76]	People	60 patients with type 2 diabetes; age 30–55 years; BMI 27 kg/m^2^–35 kg/m^2^	600 mg/d NaBut (group A), 10 g/d HP inulin (group B), 600 mg/d NaBut + 10 g/d HP inulin (Group C); placebo (group D); for 45 days	Reduction in diastolic blood pressure (in groups A, B, C) compared with the placebo group. Sodium butyrate + inulin intervention reduced fasting blood glucose levels and waist-to-hip ratio. Waist circumference (in groups B and C) decreased significantly after the intervention. Increase in GLP1 peptide levels (in groups A and C) compared to the placebo group.
N. Roshanravan et al. (2018) [72]	People	60 patients with type 2 diabetes; age 30–55 years; BMI 27 kg/m^2^–35 kg/m^2^	600 mg/d NaBut (group A), 10 g/d HP inulin (group B), 600 mg/d NaBut + 10 g/d HP inulin (Group C); placebo (group D); for 45 days	A. muciniphila increased significantly after supplementation with HP inulin and (separately) butyrate. Increased expression of microRNA-375 was observed after supplementation with butyrate and butyrate + inulin compared to the placebo group.
N. Roshanravan et al. (2020) [71]	People	60 patients with type 2 diabetes; age 30–55 years; BMI 27 kg/m^2^–35 kg/m^2^	600 mg/d NaBut (Group A), 10 g/d HP inulin (group B), 600 mg/d NaBut + 10 g/d HP inulin (Group C); placebo (group D); for 45 days	Butyrate relatively decreased the expression levels of TLR2/4, NF-κB1, caspase-1, NLRP3, IL-1β, and IL-18. Butyrate + inulin—increased fold change in miR-146a and miR-9 compared to the placebo group. Change in total antioxidant capacity and superoxide dismutase were significantly increased after butyrate and concomitant butyrate + inulin.
Z. Khosravi et al. (2022) [74]	People	42 patients with type 2 diabetes	NaBut (n = 21) (600 mg/d) or placebo (n = 21); for 6 weeks	Butyrate significantly reduced systolic and diastolic blood pressure. BS2hpp decreased significantly in the intervention group and the placebo group. NaBut significantly increased insulin levels, total cholesterol, low-density lipoprotein cholesterol and reduced NO levels.
P. Panufnik et al. (2024) [46]	People	52 patients with type 2 diabetes and abdominal pain	Butyrate at a dose of 1.5 g/day (n = 29) or placebo (n = 23); for 12 weeks	Alleviation of gastrointestinal signs and symptoms. Slight but significant improvements in BMI and HbA1C levels. A significant decrease in the incidence of SIBO.

T2DM—type 2 diabetes; NaB, NaBut—sodium butyrate; HP inulin—high-performance inulin; HbA1c—glycated haemoglobin; TC—total cholesterol; LDL-c—low-density lipoprotein cholesterol; LPS—lipopolysaccharide; Caco-2—the human colon adenocarcinoma cell line; GLP1—glucagon-lik peptide 1; BS2hpp—blood sugar level 2 h after a meal; NO—nitric oxide; BMI—body mass index; SIBO—small intestinal bacterial overgrowth.

## 8. Conclusions

Type 2 diabetes mellitus (T2DM) continues to impose an escalating global health burden, closely tied to lifestyle changes, demographic transitions, and environmental factors. Recent advances highlight the critical role of intestinal barrier integrity and gut microbiota composition in the disease’s pathogenesis. Disruption of the gut barrier leads to systemic inflammation, metabolic endotoxemia, and insulin resistance, central elements in T2DM progression. Sodium butyrate, a key microbial metabolite, exerts protective effects by strengthening the epithelial barrier function, modulating immune responses, and improving glucose metabolism. Preclinical studies provide compelling evidence for its therapeutic potential, and emerging clinical data suggest beneficial effects on glycemic control and inflammatory markers. Nevertheless, the clinical application of sodium butyrate in T2DM management remains in its early stages. Rigorous, large-scale clinical trials are needed to validate its efficacy, determine optimal dosing strategies, and establish its long-term safety profile. Targeting gut microbiota and barrier function through butyrate-based interventions may open new avenues for T2DM prevention and treatment.

It should be underlined that in light of the escalating global burden of obesity and type T2DM, it is critical to not only investigate novel therapeutic interventions, including dietary supplements (such as sodium butyrate), but also to re-evaluate the nutritional quality of modern diets. Attention should be directed toward the elimination of ultra-processed, nutrient-poor foods that offer minimal physiological benefit and may exacerbate metabolic dysfunction. Integrating these broader dietary and environmental perspectives could provide a more holistic and effective approach to managing and preventing metabolic diseases.
